# Genes and primary headaches: discovering new potential therapeutic targets

**DOI:** 10.1186/1129-2377-14-61

**Published:** 2013-07-12

**Authors:** Innocenzo Rainero, Elisa Rubino, Koen Paemeleire, Annalisa Gai, Alessandro Vacca, Paola De Martino, Salvatore Gentile, Paola Sarchielli, Lorenzo Pinessi

**Affiliations:** 1 Headache Center, Neurology I, Department of Neuroscience, University of Torino, Via Cherasco 15, Torino, 10126, Italy; 2 Department of Neurology, Ghent University Hospital, Ghent, Belgium; 3 Headache Centre, Neurologic Clinic, University of Perugia, Perugia, Italy

**Keywords:** Primary headaches, Genes, *MHTFR*, *KCNK18*, *HCRTR1*, *HCRTR2*

## Abstract

Genetic studies have clearly shown that primary headaches (migraine, tension-type headache and cluster headache) are multifactorial disorders characterized by a complex interaction between different genes and environmental factors. Genetic association studies have highlighted a potential role in the etiopathogenesis of these disorders for several genes related to vascular, neuronal and neuroendocrine functions. A potential role as a therapeutic target is now emerging for some of these genes. The main purpose of this review is to describe new advances in our knowledge regarding the role of *MTHFR*, *KCNK18, TRPV1, TRPV3* and *HCRTR* genes in primary headache disorders. Involvement of these genes in primary headaches, as well as their potential role in the therapy of these disorders, will be discussed.

## Introduction

Primary headache disorders, according to the International Classification of Headache Disorders 2^nd^ edition (ICHD-II), include migraine, tension-type headache, cluster headache, and other primary headaches [1]. Primary headaches represent a common and major health problem worldwide and significantly impair patients’ quality of life [2,3]. These disorders may affect individuals from childhood and are most troublesome in the productive years of life, thus generating an economic burden for both society and healthcare systems [4,5]. Recently, Global Burden of Disease (GBD) studies have rated primary headaches among the top ten disorders causing significant disability [6].

In recent years, genetic studies have provided substantial evidence supporting the notion that primary headaches are complex, multifactorial disorders. Population, family and twin studies have shown that migraine, tension-type headache and cluster headache have a significant heritable component [7,8]. Different genetic factors may, therefore, be involved in the generation of a specific “headache threshold”. In rare primary headache subtypes, such as Familial Hemiplegic Migraine (FHM), single gene mutations co-segregate with disease phenotype [9-11]. In the more common forms of primary headaches, as in other complex diseases, the phenotype is thought to be caused by an interaction of multiple genetic variants, each of them having a small to medium effect, with different environmental factors.

Due to the complexity of these disorders, the isolation of different genetic factors involved in primary headaches has proven to be difficult. Genetic association studies have provided evidence that genes involved in vascular, neuronal and endocrine functions may have a significant role in primary headaches [12,13]. The purpose of this review is to highlight recent discoveries, in particular about methylenetetrahydrofolate reductase (*MTHFR*), potassium channel, subfamily K member 18 (*KCNK18*), transient related potential vanilloid type 1 (TRPV1), transient related potential vanilloid type 3 (TRPV3), hypocretin (orexin) receptor 1 (*HCRTR1*) and hypocretin (orexin) receptor 2 (*HCRTR2*) genes which are involved in different subtypes of primary headaches and that, in the near future, might be of relevance as novel therapeutic targets.

## Review

In the last two decades, molecular genetic studies provided substantial evidence concerning the potential role of multiple genes in primary headaches. The majority of these studies evaluated genetic factors involved in migraine, while molecular genetics of cluster headache and tension-type headache has been little studied, so far.

### Vascular genes and primary headaches

Migraine and cluster headache have long been considered vascular disorders. Even if the so-called “vascular theory” of migraine has been shown to be inadequate in explaining the complex symptoms of the disorder, both migraineurs and cluster headache patients show abnormalities in both cranial and extracranial vascular reactivity [14,15]. In addition, both disorders are characterized by a significant comorbidity with diseases such as stroke, myocardial infarction, hypertension and Raynaud’s phenomenon [16-18]. Several genes involved in vascular functions, such as endothelin-1 (*ETA-1*), angiotensin-converting enzyme (*ACE*), Neurogenic Locus Notch Homolog Protein 4 (*NOTCH4*) and methylenetetrahydrofolate reductase (*MTHFR*) genes have been studied in patients with primary headaches and some of these, such as the *MTHFR* gene, were significantly associated with migraine [19-24].

**Figure 1:**
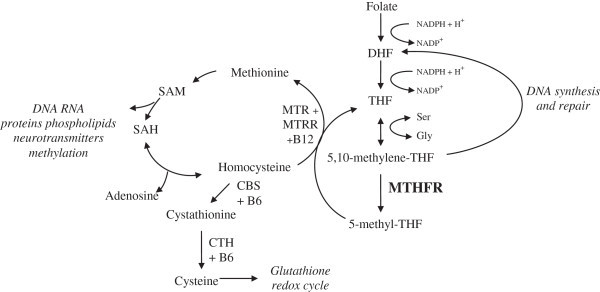
**Production of homocysteine as part of the amino acid and purine biosynthesis pathway.** DHF = dihydrofolate, THF = tetrahydrofolate, MTHFR = methylene-tetrahydrofolate-reductase, TS = thymidylate-synthase, MTR = methionine-synthase, MTRR o MSR = methionine-synthase-reductase.

The *MTHFR* gene is located on chromosome 1p36.3; it consists of 11 exons and encodes for the methylenetetrahydrofolate reductase, a crucial enzyme involved in purine and thymidylate biosynthesis, methylation of DNA and amino acids, and neurotransmitters synthesis. The MTHFR enzyme catalyzes the reduction of 5, 10-methylenetetrahydrofolate to 5-methyltetrahydrofolate, a substrate needed for the conversion of homocysteine to methionine (Figure 1). This pathway is folate-dependent and a lack of dietary folate can produce an increase in homocysteine levels. The clinical consequences of increased homocysteine plasma concentrations include endothelial cells injury and alterations in coagulant properties of blood [25-27]. Furthermore, homocysteine derivatives act as NMDA receptor agonists and they may enhance glutamatergic neurotransmission, thereby increasing the spontaneous trigeminal cells firing and predisposing cortical neurons to hyperexcitability [28,29].

Several genetic variants have been described in the *MTHFR* gene. Genetic research investigating the role of *MTHFR* in primary headaches has focused almost exclusively on two common polymorphisms, due to their functional activity. These are a cytosine (C) > thymine (T) change at position 677 in exon 4, that results in a substitution of an alanine into a valine amino acid (Ala222Val) in the catalytic domain, and an adenine (A) > cytosine (C) change occurring at position 1298 in exon 8, that changes a glutamate into an alanine amino acid (Glu429Ala). The *C677T* genetic variant allele produces a 35% reduction of MTHFR enzyme activity whereas the *A1298C* variant results in decreased MTHFR activity to a somewhat lesser degree [30]. These two variants have been extensively associated with the pathogenesis of several disorders, such as cardiovascular disease, cerebrovascular disease, and psychiatric disorders [31-35].

A large number of studies also provided evidence of an association between migraine and the C677T polymorphism in the *MTHFR* gene [22-24]. This association seems significant mainly in patients affected by migraine with aura (MA) while in patients affected by migraine without aura (MO) the results are conflicting. Two recent genetic meta-analysis provided clear evidence of a significant association between MA and the *MTHFR* gene: the carriage of the T allele was shown to be associated with an approximately two-fold increased risk [36,37].

More recently, in Norfolk Island Population, three *MTHFR* single nucleotide polymorphisms (SNPs) were associated with migraine. These three SNPs are located in intron 7 (rs6696752), in the 3′ untranslated region (rs4846048), and in exon 11 (rs2274976, non synonymous, producing a substitution of arginine to glutamine, R594Q) and have not previously been reported to show any genetic association with migraine. These findings reinforced the potential role of *MTHFR* in migraine susceptibility [37].

A recent case–control study examined the association between *MTHFR* polymorphisms and cluster headache in a group of 147 cases and 599 Caucasians controls. This study found no evidence of association between genotypes of the *MTHFR* 677C>T polymorphism and cluster headache overall. However, subgroup analyses suggested that carriers of the MTHFR 677 T allele may have an increased risk for chronic cluster headache, suggesting a need for additional studies in order to evaluate a possible role of *MHTFR* as modifier gene in the disorder [38]. At present, no study examined the potential association between tension-type headache and *MTHFR* polymorphisms.

Hyperomocysteinemia has been reported in patients with migraine [39]. Folic acid, vitamin B6 and vitamin B12 supplementation has been found to be effective in reducing the occurrence of migraine attacks [40]. Therefore, a recent pharmacogenetic study evaluated the effects of different *MTHFR* and 5-methyltetrahydrofolate-homocysteine methyltransferase reductase (*MTRR*) genotypes on the occurrence of migraine in a double-blinded placebo-controlled trial of daily vitamin B supplementation. Patients carrying the C allele of the *MTHFR* C677T variant showed a higher reduction in homocysteine levels, severity of pain and migraine disability, when compared with those with the T allele. MTRR catalyzes the remethylation of homocysteine to methionine, and, similarly, the A allele carriers of the *MTRR* A66G variants showed a higher degree of reduction in homocysteine levels, severity of pain and percentage of severe migraine disability, when compared with those carrying the GG genotypes [41]. This pivotal study suggests that both *MTHFR* and *MTRR* gene variants may influence the response to treatment with vitamin B in migraineurs. Conversely, genetic data concerning the role of vascular genes in tension-type headache are still scarce. A recent meta-analysis investigated the genetic role of the endothelin type A receptor (*EDNRA*) – one of the two receptors of the potent vasoconstrictor ETA-1 – in migraineurs and in patients with tension-type headache [42]. This meta-analysis included 440 migraineurs, 222 patients with tension-type headaches and 1323 controls from three previous studies reporting conflicting results about *EDNRA* -231G>A polymorphism. It found a significant difference in the frequency of AA genotype between migraine subjects and healthy controls. However, no differences were found in the distribution of the *EDNRA* -231G>A SNP between patients with tension-type headaches and controls.

### Neuronal genes and primary headaches

Several clinical and experimental data support the concept of abnormal cortical excitability as the pivotal physiological disturbance in migraine [43,44]. Mutations in genes that code for ion channels or pumps (*CACNA1A*, *ATP1A2*, and *SCN1A*) have been described in FHM, strongly supporting the hypothesis that migraine may be classified as a “cerebral ionopathy” [45]. Ca_V_ 2.1 (CACNA1A) calcium channels are located in the presynaptic terminal of both excitatory and inhibitory neurons, Na_V_ 1.1 (SCN1A) sodium channels are expressed in inhibitory interneurons while Na^+^/K^+^ATPase (ATP1A2) is located at the surface of glial cells (astrocytes). Knock-in mouse models carrying such mutations showed an increased susceptibility to cortical spreading depression, the likely underlying mechanism of migraine aura [46,47]. Finally, an interesting comorbidity between migraine and epilepsy has been described, further supporting a role for ion homeostasis genes in migraine pathophysiology [48,49]. However, until few years ago, no ion channel gene involvement has been described in the common form of migraine or in other primary headaches disorders.

In 2010, a frameshift mutation in the *KCNK18* gene which segregates perfectly with typical MA in a large, multigenerational pedigree was reported [50]. This gene codes for TWIK-related spinal cord potassium channel (TRESK), a member of the two-pore domain (K2P) potassium channel family. Functional characterization of the F139WfsX24 mutation demonstrated that it causes a complete loss of TRESK function and that the mutant subunit suppresses the wild-type channel function through a dominant-negative effect.

The *KCNK18* gene is located on chromosome 10; it encodes a protein containing 4 transmembrane domains (TMDs), and two pore-forming domains. The extracellular domain located between TMD1 and TMD2 contains a conserved cysteine residue that may form a disulfide bridge to aid channel dimerization, and a conserved N-linked glycosylation site, important for surface expression of the channel [51]. In humans, the family of KP2 channels includes 15 related channels but TRESK is unique in having a large intracellular regulatory domain located between TMD2 and TMD3. TRESK is abundantly expressed in the dorsal root ganglion (DRG), and in other sensory ganglia such as the trigeminal ganglion (TG). TRESK was also found in human autonomic nervous system ganglia, such as the stellate ganglion and paravertebral sympathetic chain [50,52].

The TRESK is an outwardly rectifying K^+^ current channel that contributes to the resting potential and is the most important background potassium channel in DRG. TRESK is activated in a complex manner by intracellular calcium signalling. The calcium/calmodulin-dependent protein phosphatase, calcineurin, activates TRESK function [53,54]. Calcineurin also regulates nuclear factor of activated T cells (NFATs), transcription factors that regulate inducible expression of many cytokines. In addition, recent studies have identified volatile anesthetics as highly potent TRESK agonists, interacting directly with the channel. On the contrary, cyclosporin A and tacrolimus, two potent immunosuppressants that specifically inhibit the calcineurin activation of NFATs, mimic a TRESK loss of function by keeping the channel insensitive to increases in intracellular Ca2^+^.

The physiological functions of TRESK have been mainly investigated in knock-out (KO) mice. The KO mice show no gross anatomical or behavioral phenotype. The gene ablation has minimal effect on the resting membrane potential. However, DRG neurons from TRESK KO mice displayed a lower threshold for activation, reduced action potential duration, and slightly higher amplitudes of after-hyperpolarization, suggesting that DRG neurons from KO mice were more excitable than wild-type DRG neurons [55]. In addition, due to the coupling of TRESK to the histamine H_1_ receptor, the channel may reduce neuronal excitability in inflammatory conditions when histamine or other inflammatory modulators are released into the surrounding tissue. Taken together, these data suggest an important role of TRESK in both acute and chronic pain conditions [56].

After the identification of *KCNK18* gene mutation in a Canadian MA pedigree, a large cohort of unrelated MA patients and healthy controls was screened for gene mutation. Several missense variants (R10G, A34V, C110R, S231P and A233V) were found. These variants either had no apparent functional effect, or they caused a reduction in channel activity. The A34V was identified in a single Australian migraine proband for which family samples were not available, but it was not detected in controls. By contrast, the R10G, C110R, and S231P variants were found in both migraineurs and controls. The authors concluded that the presence of a single non-functional variant in the *KCNC18* gene is probably not sufficient to determine whether an individual develops migraine [57].

In a recent study, we examined the presence of *KCNK18* gene mutations in a large data set of Italian migraine patients (both with MA and MO) and healthy controls. We confirmed the presence of *KCNK18* gene mutations in MA and also found gene mutations in MO patients [58]. Some of these gene variants have not previously been described. However, the functional relevance of these mutations still need further investigations. Finally, *KCNK18* gene involvement in tension-type headache or cluster headache has not been investigated yet.

The TRESK K2P channel is a novel and interesting component of the migraine pathogenesis pathway and represents an excellent opportunity for development of antimigraine therapy, given its highly selective expression pattern in neuronal structures, which is known to be important in disease pathogenesis. Its highly specific expression pattern in TG, DRG and parasympathetic neurons and the presumed role in abating neuronal excitability under inflammatory conditions make it an excellent target for development of new migraine therapeutics [59,60]. Specific agonists could upregulate TRESK activity and may have a potential in both acute and preventive migraine therapy, as well as in other pain disorders.

Recently, the transient receptor potential (TRP) channels gained increased interest for their potential involvement in primary headaches [61,62]. There are at least 30 members of the mammalian TRP family, which are coded by several, different genes. TRP channels are distributed in many peripheral tissues as well as central and peripheral nervous system. Several TRP family members, including the TRPV1 (Transient Related Potential Vanilloid Type 1), TRPV2 (Transient Related Potential Vanilloid Type 2), TRPV3 (Transient Related Potential Vanilloid Type 3) and TRPV4 (Transient Related Potential Vanilloid Type 4), TRPM8 (Transient Related Potential Metastatin Type 8) channels, are polymodal sensors expressed in the sensory neurons of dorsal root ganglia (DRG) and trigeminal ganglia (TG) [63]. In particular, TRPV1 receptor is highly co-expressed with calcitonin-gene related peptide (CGRP) a potent vasodilator with an important role in migraine. TRPV1 is also co-expressed with other pain signalling molecules such as substance P, P2X3 purinergic receptors and other markers of nociceptive C and Aδ fibers [64]. It has been proposed to play a crucial role as mediators of neuropathic pain and have been proposed to play a role in migraineous allodynia and sensitization phenomena [65].

Using a genetic association strategy, in 2012 Carreno et al. found a significant association between SNPs within the TRPV1 and TRPV3 genes and migraine in the Spanish population [66]. Interestingly, TRPV1 and TRPV3 are located in close proximity on the 17p13 chromosomal region and they share a high sequence homology. In the meanwhile, two genome-wide association studies (GWAS) found evidence of a significant association between genetic markers in or near the TRPM8 gene and migraine [67,68]. TRPM8 gene is expressed by a different TRPV1-negative neuronal subpopulation in DRG and TG. These preliminary data significantly support a role for TRP channels in the pathogenesis of primary headaches.

TRP channels have been among the most aggressively pursued drug targets over the past few years and several studies suggested these channels as potential therapeutic targets in migraine. Both peripheral and central nerve terminals at the spinal cord can be targeted to induce pain relief by TRPV1 agonists. In particular, the analgesic effects of the TRPV1 antagonist SB-705498 on trigeminovascular sensitization and neurotransmission have been studied in an animal model of neurovascular head pain [69,70]. Recently, a phase II clinical trial using SB-705498 has been conducted for the acute treatment of migraine attacks but results are pending (ClinicalTrials.gov).

### Neuroendocrine genes and primary headaches

A large number of endocrine abnormalities has been described in patients with primary headaches [71,72]. The hypothalamus, with its paramount control of the endocrine system, as well as its widespread connections with both central and autonomic nervous system, exerts a pivotal role in the pathogenesis of both migraine and cluster headache [73]. Therefore, genes that code for proteins involved in endocrine functions are candidate genes for primary headache disorders. Polymorphisms in genes that code for estrogen and progesterone receptors have been intensively studied in migraineurs, with contrasting results [74,75].

In 1998, two research groups independently discovered a new hypothalamic peptidergic system. The former group named the peptides “hypocretins”, because of their hypothalamic location and structural similarity to the incretin family of hormones [76]. The latter group named the peptides “orexins”, due to the appetite-enhancing properties when administered centrally to rats [77].

Subsequent studies revealed complex and interesting neurobiological effects of these peptides, with particular relevance to the pathophysiology of primary headaches [78].

The hypocretins (Hcrt-1 and Hcrt-2), also called orexins, are peptides derived by proteolytic cleavage from the same 130 amino acid precursor peptide (prepro-hypocretin). A single gene located on chromosome 17q21 in humans is responsible for encoding prepro-hypocretin. The human prepro-hypocretin gene consists of 2 exons and 1 intron. The hypocretins bind to 2 G-protein coupled receptors, termed HCRTR1 and HCRTR2. The *HCRTR1* gene in humans is located on chromosome1p33 whereas the *HCRTR2* gene is located on chromosome 6p11. Both genes consist of 7 exons and 6 introns. Hcrt-1 has equal affinity for both HCRTR1 and HCRTR2, with Hcrt-2 demonstrating a 10-fold higher affinity for HCRTR2 than HCRTR1. Activation of both receptors results in elevated levels of the intracellular Ca^2+^ concentrations, and this in turn results in the enhancement of the Gq-mediated stimulation of phospholipase C. Hypocretin immunoreactive cell bodies have been observed mainly in the hypothalamus [79]. Hypocretin-containing neurons have widespread projections throughout the CNS with particularly dense excitatory projections to monoaminergic and serotonergic brainstem centers [80]. The hypocretin system influences a wide range of physiological processes in mammals, such as feeding, arousal, rewards, and drug addiction [81,82]. Recently, a number of studies in experimental animals showed that hypocretins are involved in pain modulation within the CNS, and suggested an important role for these peptides in primary headaches [83].

In 2004, our research group investigated the possible involvement of the hypocretin transmission in cluster headache. We selected several DNA polymorphisms of the three genes that constitute the hypocretin system and, using a case–control strategy, we evaluated possible allelic and genotypic differences in a group of 109 CH patients and 211 controls. Genetic analysis revealed that both allelic and genotypic frequencies of the G1246A polymorphism in the *HCRTR2* gene were significantly different between CH patients and controls [84]. Subjects homozygous for the G allele, in comparison with the remaining genotypes, were 5-fold more likely to develop the disease. This association was confirmed in a large group of CH patients and controls from Germany [85]. On the contrary, Baumber et al. found no association between CH and the *HCRTR2* gene in a cohort of 259 patients of Danish, Swedish, and British origin [86]. To resolve this issue, we performed a genetic meta-analysis of the previous studies (593 cases and 599 controls) and a haplotype analysis: both these studies confirmed the presence of a significant association between the *HCRTR2* gene and CH [87]. At present, the possible involvement of the hypocretin system in migraine has been scarcely investigated. Studies in CH patients prompted two independent research groups to evaluate the association of the G1246A polymorphisms in the *HCRTR2* gene with migraine. Both studies found no association between this polymorphism and migraine or its clinical subtypes [88,89]. Recently, we performed a genetic case–control study to investigate whether genetic variants in the *HCRTR1* gene could modify the occurrence and the clinical features of migraine. Using a case–control strategy we genotyped 384 migraine patients and 259 controls for three SNPs in the *HCRTR1* gene. Genotypic and allelic frequencies of the rs2271933 non-synonymous polymorphism were different between migraineurs and controls [90]. The carriage of the A allele was associated with an increased migraine risk. This study supports the hypothesis that the *HCRTR1* gene could represent a genetic susceptibility factor for migraine and suggests that the hypocretin system may have a role also in the pathophysiology of migraine. Unfortunately, no data regarding involvement of *HCRTR* genes in tension-type headache are currently available.

The growing knowledge concerning the role of hypocretins/orexins in different neurological conditions has generated considerable interest in developing small-molecule hypocretin receptor antagonists as a novel therapeutic strategy. Hypocretin antagonists, especially those that block Hcrtr1 or both Hcrtr1 and Hcrtr2 receptors, have been studied mainly as new drugs for sleep disorders. In experimental animals hypocretin/orexin antagonists (almorexant, suvorexant) clearly promote sleep, and clinical results are encouraging [91-94]. Considering the high frequency of sleep disorders occurring in patients with migraine and CH, these drugs offer a new perspective in the treatment of these disorders. Finally, a pivotal role for these peptides in drug reward and drug seeking has been established and their potential role as anti-relapse medication in drug addiction is currently under investigation in experimental animals [95].

## Conclusions

The main goal of genetic studies is to unravel molecular pathways underlying primary headache disorders, in order to discover new therapeutic targets. Recent studies have highlighted a potential role for new genes, like *MTHFR*, *KCNK18, TRPV1, TRPV3*, and new neurotransmission systems, like the hypocretin system, both in migraine and cluster headache. Additional experimental and clinical studies are needed to better elucidate the involvement of these new genes in primary headaches and to evaluate new therapeutic strategies.

## Competing interest

The authors declare no competing interest regarding this manuscript.

## Authors’ contribution

IR and ER planned the review and wrote the manuscript, with input from other authors. KP reviewed the manuscript. AG and AV performed bibliographic research and draw the pictures. PDM, SG, PS and LP supervised the project. All authors read and approved the final manuscript.

## References

[B1] Headache Classification Subcommittee of the International Headache Society The international classification of headache disorders: 2nd edn Cephalalgia 2004 14 Suppl 1 9 160 14979299 10.1111/j.1468-2982.2003.00824.x

[B2] Lantéri-Minet M Duru G Mudge M Cottrell S Quality of life impairment, disability and economic burden associated with chronic daily headache, focusing on chronic migraine with or without medication overuse: a systematic review Cephalalgia 2011 14 7 837 850 10.1177/0333102411398400 21464078

[B3] Raggi A Giovannetti AM Quintas R D’Amico D Cieza A Sabariego C Bickenbach JE Leonardi M A systematic review of the psychosocial difficulties relevant to patients with migraine J Headache Pain 2012 14 8 595 606 10.1007/s10194-012-0482-1 23001069 PMC3484254

[B4] Pradalier A Auray JP El Hasnaoui A Alzahouri K Dartigues JF Duru G Henry P Lantéri-Minet M Lucas C Chazot G Gaudin AF Economic impact of migraine and other episodic headaches in France: data from the GRIM2000 study PharmacoEconomics 2004 14 15 985 999 10.2165/00019053-200422150-00003 15449963

[B5] Bloudek LM Stokes M Buse DC Wilcox TK Lipton RB Goadsby PJ Varon SF Blumenfeld AM Katsarava Z Pascual J Lanteri-Minet M Cortelli P Martelletti P Cost of healthcare for patients with migraine in five European countries: results from the International Burden of Migraine Study (IBMS) J Headache Pain 2012 14 5 361 378 10.1007/s10194-012-0460-7 22644214 PMC3381065

[B6] Vos T Flaxman AD Naghavi M Lozano R Michaud C Ezzati M Years lived with disability (YLD) for 1160 sequelae of 289 diseases and injuries 1990–2010: a systemati c analysis for the Global Burden of Disease Study 2010 Lancet 2012 14 9859 2163 2196 10.1016/S0140-6736(12)61729-2 23245607 PMC6350784

[B7] Russell MB Genetics in primary headaches J Headache Pain 2007 14 3 190 195 10.1007/s10194-007-0389-4 17563838 PMC2780622

[B8] Svensson DA Larsson B Waldenlind E Pedersen NL Shared rearing environment in migraine: results from twins reared apart and twins reared together Headache 2003 14 3 235 244 10.1046/j.1526-4610.2003.03047.x 12603642

[B9] Ophoff RA Terwindt GM Vergouwe MN Familial hemiplegic migraine and episodic ataxia type-2 are caused by mutations in the Ca2+ channel gene CACNL1A4 Cell 1996 14 543 552 10.1016/S0092-8674(00)81373-2 8898206

[B10] De Fusco M Marconi R Silvestri L Haploinsufficiency of ATP1A2 encoding the Na+/K+ pump alpha2 subunit associated with familial hemiplegic migraine type 2 Nat Genet 2003 14 192 196 10.1038/ng1081 12539047

[B11] Dichgans M Freilinger T Eckstein G Mutation in the neuronal voltage-gated sodium channel SCN1A in familial hemiplegic migraine Lancet 2005 14 371 377 10.1016/S0140-6736(05)66786-4 16054936

[B12] Colson NJ Lea RA Quinlan S Griffiths LR The role of vascular and hormonal genes in migraine susceptibility Mol Genet Metab 2006 14 107 113 10.1016/j.ymgme.2005.11.013 16403664

[B13] Maher BH Griffiths LR Identification of molecular genetic factors that influence migraine Mol Genet Genomics 2011 14 6 433 446 10.1007/s00438-011-0622-3 21519858

[B14] Vanmolkot FH Van Bortel LM de Hoon JN Altered arterial function in migraine of recent onset Neurology 2007 14 19 1563 1570 10.1212/01.wnl.0000260964.28393.ed 17460157

[B15] Barriga FJ Cuadrado ML Bueno A Barón M Dobato JL Vela L Pareja JA Clus ter headache: orbital hemodynamic changes during Valsalva maneuver Headache 2006 14 2 298 305 10.1111/j.1526-4610.2006.00287.x 16492239

[B16] Kurth T The association of migraine with ischemic stroke Curr Neurol Neurosci Rep 2010 14 2 133 139 10.1007/s11910-010-0098-2 20425238

[B17] Kurth T Gaziano JM Cook NR Logroscino G Diener HC Buring JE Migraine and risk of cardiovascular disease in women JAMA 2006 14 3 283 291 10.1001/jama.296.3.283 16849661

[B18] Sacco S Ricci S Carolei A Migraine and vascular diseases: a review of the evidence and potential implications for management Cephalalgia 2012 14 10 785 795 10.1177/0333102412451361 22711902

[B19] Tzourio C El Amrani M Poirier O Nicaud V Bousser MG Alpérovitch A Association between migraine and endothelin type A receptor (ETA −231 A/G) gene polymorphism Neurology 2001 14 10 1273 1277 10.1212/WNL.56.10.1273 11376172

[B20] Tronvik E Stovner LJ Schrader H Bovim G Involvement of the renin-angiotensin system in migraine J Hypertens 2006 14 139 143 10.1097/01.hjh.0000220419.86149.11 16601567

[B21] Rubino E Fenoglio P Gallone S Govone F Vacca A De Martino P Giobbe ML Boschi S Pinessi L Gentile S Rainero I Genetic variants in the NOTCH4 gene influence the clinical features of migraine J Headache Pain 2013 14 1 28 10.1186/1129-2377-14-28 23566281 PMC3620438

[B22] Scher AI Terwindt GM Verschuren WM Kruit MC Blom HJ Kowa H Frants RR van den Maagdenberg AM van Buchem M Ferrari MD Launer LJ Migraine and MTHFR C677T genotype in a population-based sample Ann Neurol 2006 14 2 372 375 10.1002/ana.20755 16365871

[B23] Liu A Menon S Colson NJ Quinlan S Cox H Peterson M Tiang T Haupt LM Lea RA Griffiths LR Analysis of the MTHFR C677T variant with migraine phenotypes BMC Res Notes 2010 14 213 10.1186/1756-0500-3-213 20663228 PMC2919563

[B24] Samaan Z Gaysina D Cohen-Woods S Craddock N Jones L Korszun A Owen M Mente A McGuffin P Farmer A Methylenetetrahydrofolate reductase gene variant (MTHFR C677T) and migraine: a case control study and meta-analysis BMC Neurol 2011 14 66 10.1186/1471-2377-11-66 21635773 PMC3120667

[B25] McCully KS Chemical pathology of homocysteine. IV. Excitotoxicity, oxidative stress, endothelial dysfunction, and inflammation Ann Clin Lab Sci 2009 14 3 219 232 19667406

[B26] Dionisio N Jardín I Salido GM Rosado JA Homocysteine, intracellular signaling and thrombotic disorders Curr Med Chem 2010 14 27 3109 3119 10.2174/092986710791959783 20629621

[B27] Boldyrev A Bryushkova E Mashkina A Vladychenskaya E Why is Homocysteine toxic for the nervous and immune systems? Curr Aging Sci 2012 [Epub ahead of print] 10.2174/18746098112059990007 23237596

[B28] Zieminska E Lazarewicz JW Excitotoxic neuronal injury in chronic homocysteine neurotoxicity studied in vitro: the role of NMDA and group I metabotropic glutamate receptors Acta Neurobiol Exp (Wars) 2006 14 4 301 309 17265692 10.55782/ane-2006-1619

[B29] Yeganeh F Nikbakht F Bahmanpour S Rastegar K Namavar R Neuroprotective effects of NMDA and Group I metabotropic glutamate receptor antagonists against neurodegeneration induced by homocysteine in rat hippocampus: in vivo study J Mol Neurosci 2013 [Epub ahead of print] 10.1007/s12031-013-9996-5 23564299

[B30] Frosst P Blom HJ Milos R Goyette P Sheppard CA Matthews RG Boers GJ den Heijer M Kluijtmans LA van den Heuvel LP Rozen R A candidate genetic risk factor for vascular disease: a common mutation in methylenetetrahydrofolate reductase Nat Genet 1995 14 1 111 113 10.1038/ng0595-111 7647779

[B31] Trabetti E Homocysteine, MTHFR gene polymorphisms, and cardio-cerebrovascular risk J Appl Genet 2008 14 3 267 282 10.1007/BF03195624 18670064

[B32] Pizza V Bisogno A Lamaida E Agresta A Bandieramonte G Volpe A Galasso R Galasso L Caputo M Tecce MF Capasso A Migraine and coronary artery disease: an open study on the genetic polymorphism of the 5, 10 methylenetetrahydrofolate (MTHFR) and angiotensin I-converting enzyme (ACE) genes Cent Nerv Syst Agents Med Chem 2010 14 2 91 96 10.2174/187152410791196404 20518725

[B33] Sazci A Ergul E Tuncer N Akpinar G Kara I Methylenetetrahydrofolate reductase gene polymorphisms are associated with ischemic and hemorrhagic stroke: dual effect of MTHFR polymorphisms C677T and A1298C Brain Res Bull 2006 14 1–3 45 50 17113927 10.1016/j.brainresbull.2006.07.014

[B34] Almeida OP McCaul K Hankey GJ Norman P Jamrozik K Flicker L Homocysteine and depression in later life Arch Gen Psychiatry 2008 14 11 1286 1294 10.1001/archpsyc.65.11.1286 18981340

[B35] Peerbooms OL van Os J Drukker M Kenis G Hoogveld L de Hert M Delespaul P van Winkel R Rutten BP MTHFR in Psychiatry Group Meta-analysis of MTHFR gene variants in schizophrenia, bipolar disorder and unipolar depressive disorder: evidence for a common genetic vulnerability? Brain Behav Immun 2011 14 8 1530 1543 10.1016/j.bbi.2010.12.006 21185933

[B36] Rubino E Ferrero M Rainero I Binello E Vaula G Pinessi L Association of the C677T polymorphism in the MTHFR gene with migraine: a meta-analysis Cephalalgia 2009 14 818 825 17714520 10.1111/j.1468-2982.2007.01400.x

[B37] Schürks M Rist PM Kurth T MTHFR 677C>T and ACE D/I polymorphisms in migraine: a systematic review and meta-analysis Headache 2010 14 4 588 599 10.1111/j.1526-4610.2009.01570.x 19925624 PMC3071567

[B38] Schürks M Neumann FA Kessler C Diener HC Kroemer HK Kurth T Völzke H Rosskopf D MTHFR 677C>T polymorphism and cluster headache Headache 2010 14 2 201 207 20946434 10.1111/j.1526-4610.2010.01780.x

[B39] Moschiano F D’Amico D Usai S Grazzi L Di Stefano M Ciusani E Erba N Bussone G Homocysteine plasma levels in patients with migraine with aura Neurol Sci 2008 14 Suppl 1 S173 S175 18545927 10.1007/s10072-008-0917-2

[B40] Lea R Colson N Quinlan S Macmillan J Griffiths L The effects of vitamin supplementation and MTHFR (C677T) genotype on homocysteine-lowering and migraine disability Pharmacogenet Genomics 2009 14 6 422 428 10.1097/FPC.0b013e32832af5a3 19384265

[B41] Menon S Lea RA Roy B Hanna M Wee S Haupt LM Oliver C Griffiths LR Genotypes of the MTHFR C677T and MTRR A66G genes act independently to reduce migraine disability in response to vitamin supplementation Pharmacogenet Genomics 2012 14 10 741 749 10.1097/FPC.0b013e3283576b6b 22926161

[B42] Miao J Wang F Fang Y Association of 231G>A polymorphism of endothelin type A receptor gene with migraine: a meta-analysis J Neurol Sci 2012 14 1–2 232 235 23058564 10.1016/j.jns.2012.09.027

[B43] Haigh S Karanovic O Wilkinson F Wilkins A Cortical hyperexcitability in migraine and aversion to patterns Cephalalgia 2012 14 3 236 240 10.1177/0333102411433301 22234882 PMC4011802

[B44] Coppola G Pierelli F Schoenen J Is the cerebral cortex hyperexcitable or hyperresponsive in migraine? Cephalalgia 2007 14 12 1427 1439 10.1111/j.1468-2982.2007.01500.x 18034686

[B45] Ferrari MD van der Maagdenberg AM Frants RR Goadsby PJ Waxman SG Migraine as a cerebral ionopathy with impaired central sensory processing Molecular Neurology 2007 Amsterdam: Elsevier 439 461

[B46] Eikermann-Haerter K Yuzawa I Qin T Wang Y Baek K Kim YR Hoffmann U Dilekoz E Waeber C Ferrari MD van den Maagdenberg AM Moskowitz MA Ayata C Enhanced subcortical spreading depression in familial hemiplegic migraine type 1 mutant mice J Neurosci 2011 14 15 5755 5763 10.1523/JNEUROSCI.5346-10.2011 21490217 PMC3135337

[B47] Leo L Gherardini L Barone V De Fusco M Pietrobon D Pizzorusso T Casari G Increased susceptibility to cortical spreading depression in the mouse model of familial hemiplegic migraine type 2 PLoS Genet 2012 14 6 e1002129 10.1371/journal.pgen.1002129 PMC3121757 21731499

[B48] Bianchin MM Londero RG Lima JE Bigal ME Migraine and epilepsy: a focus on overlapping clinical, pathophysiological, molecular, and therapeutic aspects Curr Pain Headache Rep 2010 14 4 276 283 10.1007/s11916-010-0121-y 20495966

[B49] Striano P Belcastro V Verrotti A Parisi P “Comorbidity” between epilepsy and headache/migraine: the other side of the same coin! J Headache Pain 2011 14 5 577 578 10.1007/s10194-011-0371-z 21805357 PMC3173652

[B50] Lafrenière RG Cader MZ Poulin JF Andres-Enguix I Simoneau M Gupta N Boisvert K Lafrenière F McLaughlan S Dubé MP Marcinkiewicz MM Ramagopalan S Ansorge O Brais B Sequeiros J Pereira-Monteiro JM Griffiths LR Tucker SJ Ebers G Rouleau GA A dominant-negative mutation in the TRESK potassium channel is linked to familial migraine with aura Nat Med 2010 14 10 1157 1160 10.1038/nm.2216 20871611

[B51] Egenberger B Polleichtner G Wischmeyer E Döring F N-linked glycos ylation determines cell surface expression of two-pore-domain K+ channel TRESK Biochem Biophys Res Commun 2010 14 2 1262 1267 10.1016/j.bbrc.2009.12.056 20006580

[B52] Kang D Mariash E Kim D Functional expression of TRESK-2, a new member of the tandem-pore K+ channel family J Biol Chem 2004 14 27 28063 28070 10.1074/jbc.M402940200 15123670

[B53] Li H Rao A Hogan PG Structural delineation of the calcineurin–NFAT inte raction and its parallels to PP1 targeting interactions J Mol Biol 2004 14 5 1659 1674 10.1016/j.jmb.2004.07.068 15364589

[B54] Enyedi P Braun G Czirják G TRESK: the lone ranger of two-pore domain potassium channels Mol Cell Endocrinol 2012 14 1–2 75 81 22115960 10.1016/j.mce.2011.11.009

[B55] Dobler T Springauf A Tovornik S Weber M Schmitt A Sedlmeier R Wischmeyer E Döring F TRESK two-pore-domain K+ channels constitute a significant component of background potassium currents in murine dorsal root ganglion neurones J Physiol 2007 14 Pt 3 867 879 17962323 10.1113/jphysiol.2007.145649 PMC2375503

[B56] Huang DY Yu BW Fan QW Roles of TRESK, a novel two-pore domain K+ channel, in pain pathway and general anesthesia Neurosci Bull 2008 14 3 166 172 10.1007/s12264-008-0225-0 18500390 PMC5552547

[B57] Andres-Enguix I Shang L Stansfeld PJ Morahan JM Sansom MS Lafrenière RG Roy B Griffiths LR Rouleau GA Ebers GC Cader ZM Tucker SJ Functional analysis of missense variants in the TRESK (KCNK18) K channel Sci Rep 2012 14 237 240 22355750 10.1038/srep00237 PMC3266952

[B58] Rainero I Rubino E Fenoglio P Gallone S Zavarise P Carli D Boschi S Gai A Pinessi L Dalla Volta G Investigation of KCNK18 (TRESK) Genetic Variants in Migraine with and without Aura. In: Abstracts of the 65th AAN Annual Meeting, San Diego Neurology 2013 14 Meeting Abstracts 1 S55.003

[B59] Marsh B Acosta C Djouhri L Lawson SN Leak K^+^ channel mRNAs in dorsal root ganglia: relation to inflammation and spontaneous pain behaviour Mol Cell Neurosci 2012 14 3 375 386 10.1016/j.mcn.2012.01.002 22273507 PMC3334831

[B60] Tulleuda A Cokic B Callejo G Saiani B Serra J Gasull X TRESK channel contribution to nociceptive sensory neurons excitability: modulation by nerve injury Mol Pain 2011 14 30 10.1186/1744-8069-7-30 21527011 PMC3095542

[B61] Nassini R De Cesaris F Pedretti P Geppetti P TRPS and migraine The Open Drug Discovery Journal 2010 14 55 63 10.2174/1877381801002030055

[B62] Oxford GS Hurley JH The role of TRP channels in migraine The Open Pain Journal 2013 14 Suppl 1 37 49

[B63] Vennekens R Menigoz A Nilius B TRPs in the Brain Rev Physiol Biochem Pharmacol 2012 14 27 64 23184016 10.1007/112_2012_8

[B64] Bae YC Oh JM Hwang SJ Shigenaga Y Valtschanoff JG Expression of vanilloid receptor TRPV1 in the rat trigeminal sensory nuclei J Comp Neurol 2004 14 1 62 71 10.1002/cne.20272 15334649

[B65] Meents JE Neeb L Reuter U TRPV1 in migraine pathophysiology Trends Mol Med 2010 14 4 153 159 10.1016/j.molmed.2010.02.004 20347391

[B66] Carreño O Corominas R Fernández-Morales J Camiña M Sobrido MJ Fernández-Fernández JM Pozo-Rosich P Cormand B Macaya A SNP variants within the vanilloid TRPV1 and TRPV3 receptor genes are associated with migraine in the Spanish population Am J Med Genet B Neuropsychiatr Genet 2012 14 1 94 103 10.1002/ajmg.b.32007 22162417

[B67] Chasman DI Schürks M Anttila V de Vries B Schminke U Launer LJ Terwindt GM van den Maagdenberg AM Fendrich K Völzke H Ernst F Griffiths LR Buring JE Kallela M Freilinger T Kubisch C Ridker PM Palotie A Ferrari MD Hoffmann W Zee RY Kurth T Genome-wide association study reveals three susceptibility loci for common migraine in the general population Nat Genet 2011 14 7 695 698 10.1038/ng.856 21666692 PMC3125402

[B68] Freilinger T Anttila V de Vries B Malik R Kallela M Terwindt GM Pozo-Rosich P Winsvold B Nyholt DR van Oosterhout WP Artto V Todt U Hämäläinen E Fernández-Morales J Louter MA Kaunisto MA Schoenen J Raitakari O Lehtimäki T Vila-Pueyo M Göbel H Wichmann E Sintas C Uitterlinden AG Hofman A Rivadeneira F Heinze A Tronvik E van Duijn CM Kaprio J Genome-wide association analysis identifies susceptibility loci for migraine without aura Nat Genet 2012 14 7 777 782 10.1038/ng.2307 22683712 PMC3773912

[B69] Lambert GA Davis JB Appleby JM Chizh BA Hoskin KL Zagami AS The effects of the TRPV1 receptor antagonist SB-705498 on trigeminovascular sensitisation and neurotransmission Naunyn Schmiedebergs Arch Pharmacol 2009 14 4 311 325 10.1007/s00210-009-0437-5 19690836

[B70] Szallasi A Cruz F Geppetti P RPV1: a therapeutic target for novel analgesic drugs? Trends Mol Med 2006 14 11 545 554 10.1016/j.molmed.2006.09.001 16996800

[B71] Silberstein SD The role of sex hormones in headache Neurology 1992 14 Suppl 2 37 42 1557190

[B72] Nappi RE Nappi G Neuroendocrine aspects of migraine in women Gynecol Endocrinol 2012 14 Suppl 1 37 41 22394302 10.3109/09513590.2012.651931

[B73] Alstadhaug KB Migraine and the hypothalamus Cephalalgia 2009 14 8 809 817 10.1111/j.1468-2982.2008.01814.x 19604254

[B74] Schürks M Rist PM Kurth T Sex hormone receptor gene polymorphisms and migrain e: a systematic review and meta-analysis Cephalalgia 2010 14 1306 1328 20959426 10.1177/0333102410364155 PMC3055237

[B75] Joshi G Pradhan S Mittal B Role of the oestrogen receptor (ESR1 PvuII and ESR1 325 C->G) and progesterone receptor (PROGINS) polymorphisms in genetic susceptibility to migraine in a North Indian population Cephalalgia 2010 14 3 311 320 19673915 10.1111/j.1468-2982.2009.01967.x

[B76] De Lecea L Kilduff TS Peyron C Gao X Foye PE Danielson PE Fukuhara C Battenberg EL Gautvik VT Bartlett FS 2nd Frankel WN van den Pol AN Bloom FE Gautvik KM Sutcliffe JG The hypocretins: hypothalamus-specific peptides with neuroexcitatory activity Proc Natl Acad Sci USA 1998 14 1 322 327 10.1073/pnas.95.1.322 9419374 PMC18213

[B77] Sakurai T Amemiya A Ishii M Matsuzaki I Chemelli RM Tanaka H Williams SC Richardson JA Kozlowski GP Wilson S Arch JR Buckingham RE Haynes AC Carr SA Annan RS McNulty DE Liu WS Terrett JA Elshourbagy NA Bergsma DJ Yanagisawa M Orexins and orexin receptors: a family of hypothalamic neuropeptides and G protein-coupled receptors that regulate feeding behavior Cell 1998 14 4 573 585 10.1016/S0092-8674(00)80949-6 9491897

[B78] Rainero I De Martino P Pinessi L Hypocretins and primary headaches: neurobiology and clinical implications Expert Rev Neurother 2008 14 3 409 416 10.1586/14737175.8.3.409 18345971

[B79] Nishino S The hypothalamic peptidergic system, hypocretin/orexin and vigilance control Neuropeptides 2007 14 3 117 133 10.1016/j.npep.2007.01.003 17376528

[B80] Mori T Ito S Kuwaki T Yanagisawa M Sakurai T Sawaguchi T Monoaminergic neuronal changes in orexin deficient mice Neuropharmacology 2012 14 4–5 826 832 10.1016/j.neuropharm.2009.08.009 19703479

[B81] Girault EM Yi CX Fliers E Kalsbeek A Orexins, feeding, and energy balance Prog Brain Res 2012 14 47 64 22813969 10.1016/B978-0-444-59489-1.00005-7

[B82] Mahler SV Smith RJ Moorman DE Sartor GC Aston-Jones G Multiple roles for orexin/hypocretin in addiction Prog Brain Res 2012 14 79 121 22813971 10.1016/B978-0-444-59489-1.00007-0 PMC3643893

[B83] Yamamoto T Saito O Shono K Aoe T Chiba T Anti-mechanical allodynic effect of intrathecal and intracerebroventricular injection of orexin-A in the rat neuropathic pain model Neurosci Lett 2003 14 3 183 186 10.1016/S0304-3940(03)00716-X 12875916

[B84] Rainero I Gallone S Valfrè W Ferrero M Angilella G Rivoiro C Rubino E De Martino P Savi L Ferrone M Pinessi L A polymorphism of the hypocretin receptor 2 gene is associated with cluster headache Neurology 2004 14 7 1286 1288 10.1212/01.WNL.0000142424.65251.DB 15477554

[B85] Schürks M Kurth T Geissler I Tessmann G Diener HC Rosskopf D Cluster heada che is associated with the G1246A polymorphism in the hypocretin receptor 2 gene Neurology 2006 14 12 1917 1919 10.1212/01.wnl.0000215852.35329.34 16554494

[B86] Baumber L Sjöstrand C Leone M Harty H Bussone G Hillert J Trembath RC MB R e A genome-wide scan and HCRTR2 candidate gene analysis in a European cluster headache cohort Neurology 2006 14 12 1888 1893 10.1212/01.wnl.0000219765.95038.d7 16801656

[B87] Rainero I Rubino E Valfrè W Gallone S De Martino P Zampella E Pinessi L Association between the G1246A polymorphism of the hypocretin receptor 2 gene and cluster headache: a meta-analysis J Headache Pain 2007 14 3 152 156 10.1007/s10194-007-0383-x 17563843 PMC3476142

[B88] Pinessi L Binello E De Martino P Gallone S Gentile S Rainero I Rivoiro C Rubino E Savi L Valfrè W Vaula G The 1246G–>A polymorphism of the HCRTR2 gene is not associated with migraine Cephalalgia 2007 14 8 945 949 10.1111/j.1468-2982.2007.01347.x 17645762

[B89] Schürks M Limmroth V Geissler I Tessmann G Savidou I Engelbergs J Kurth T Di ener HC Rosskopf D Association between migraine and the G1246A polymorphism in the hypocretin receptor 2 gene Headache 2007 14 8 1195 1199 10.1111/j.1526-4610.2007.00863.x 17883525

[B90] Rainero I Rubino E Gallone S Fenoglio P Picci LR Giobbe L Ostacoli L Pinessi L Evidence for an association between migraine and the hypocretin receptor 1 gene J Headache Pain 2011 14 2 193 199 10.1007/s10194-011-0314-8 21344296 PMC3072499

[B91] Inutsuka A Yamanaka A The physiological role of orexin/hypocretin neurons in the regulation of sleep/wakefulness and neuroendocrine functions Front Endocrinol (Lausanne) 2013 14 18 23508038 10.3389/fendo.2013.00018 PMC3589707

[B92] Mang GM Dürst T Bürki H Imobersteg S Abramowski D Schuepbach E Hoyer D F endt M Gee CE The dual orexin receptor antagonist almorexant induces sleep and decreases orexin- induced locomotion by blocking orexin 2 receptors Sleep 2012 14 12 1625 1635 23204605 10.5665/sleep.2232 PMC3490355

[B93] Sun H Kennedy WP Wilbraham D Lewis N Calder N Li X Ma J Yee KL Ermlich S Mangin E Lines C Rosen L Chodakewitz J Murphy GM Effects of suvorexant, an orexin receptor antagonist, on sleep parameters as measured by polysomnography in healthy men Sleep 2013 14 2 259 267 23372274 10.5665/sleep.2386 PMC3542986

[B94] Mieda M Sakurai T Orexin (hypocretin) receptor agonists and antagonists for treatment of sleep disorders. Rationale for development and current status CNS Drugs 2013 14 2 83 90 10.1007/s40263-012-0036-8 23359095

[B95] Zhou L Ghee SM Chan C Lin L Cameron MD Kenny PJ See RE Orexin-1 receptor mediation of cocaine seeking in male and female rats J Pharmacol Exp Ther 2012 14 3 801 809 10.1124/jpet.111.187567 22186370 PMC3286310

